# Gene expression during the formation of resting spores induced by nitrogen starvation in the marine diatom *Chaetoceros socialis*

**DOI:** 10.1186/s12864-023-09175-x

**Published:** 2023-03-10

**Authors:** Angela Pelusi, Luca Ambrosino, Marco Miralto, Maria Luisa Chiusano, Alessandra Rogato, Maria Immacolata Ferrante, Marina Montresor

**Affiliations:** 1 Department of Integrative Marine Ecology, Stazione Zoologica Anton Dohrn, Villa Comunale, 80121 Naples, Italy; 2 Department of Research Infrastructures for Marine Biological Resources, Stazione Zoologica Anton Dohrn, Villa Comunale, 80121 Naples, Italy; 3 Department of Agricultural Sciences, University of Naples “Federico II”, Via Università 100, 80055 Portici, Naples, Italy; 4 Institute of Bioscience and BioResources, IBBR- CNR, Via Pietro Castellino 111, 80131 Naples, Italy; 5 Oceanography Division, National Institute of Oceanography and Applied Geophysics – OGS, Via Auguste Piccard, 54, 34151 Trieste, Italy

**Keywords:** Transcriptomics, Resting stages, Nitrogen transporters, Nitrogen starvation, Diatoms, *Chaetoceros socialis*, Cell signaling, Lipoxygenase, PCD

## Abstract

**Background:**

Dormancy is widespread in both multicellular and unicellular organisms. Among diatoms, unicellular microalgae at the base of all aquatic food webs, several species produce dormant cells (spores or resting cells) that can withstand long periods of adverse environmental conditions.

**Results:**

We present the first gene expression study during the process of spore formation induced by nitrogen depletion in the marine planktonic diatom *Chaetoceros socialis*. In this condition, genes related to photosynthesis and nitrate assimilation, including high-affinity nitrate transporters (*NTRs),* were downregulated. While the former result is a common reaction among diatoms under nitrogen stress, the latter seems to be exclusive of the spore-former *C. socialis.* The upregulation of catabolic pathways, such as tricarboxylic acid cycle, glyoxylate cycle and fatty acid beta-oxidation, suggests that this diatom could use lipids as a source of energy during the process of spore formation. Furthermore, the upregulation of a lipoxygenase and several aldehyde dehydrogenases (*ALDHs*) advocates the presence of oxylipin-mediated signaling, while the upregulation of genes involved in dormancy-related pathways conserved in other organisms (e.g. serine/threonine-protein kinases *TOR* and its inhibitor *GATOR*) provides interesting avenues for future explorations.

**Conclusions:**

Our results demonstrate that the transition from an active growth phase to a resting one is characterized by marked metabolic changes and provides evidence for the presence of signaling pathways related to intercellular communication.

**Supplementary Information:**

The online version contains supplementary material available at 10.1186/s12864-023-09175-x.

## Background

All over the phylogenetic tree of life, many organisms can undergo a dormant phase in which they stop the developmental program or the vegetative division cycle for a variable length of time in response to unfavourable conditions for growth. Unicellular species, both prokaryotes and eukaryotes, produce resting stages such as akinetes, cysts, or spores, characterized by markedly reduced metabolism, increased content of storage material and often considerable morphological modifications of the cell wall ([1, 2]). The reversible switch from an active growth phase to a resting one is generally induced by the perception of specific environmental signals, such as changes in temperature, photoperiod or concentration of nutrients. The transition between life cycle phases implies energetic investments and dramatic changes in several metabolic pathways ([1]); however, the molecular mechanisms at the base of this transition are still far to be elucidated and have been mainly explored for a few model species and cell types of medical interest (e.g., [3]).

Diatoms are a very diverse group of unicellular microalgae, at the base of aquatic food web and key players in different biogeochemical cycles ([4, 5]). Several species produce resting stages that can be either morphologically differentiated spores or resting cells, apparently indistinguishable from the vegetative ones ([6]). Resting stages are characterized by high carbon (C) content and can be responsible for considerable C export to the benthic compartment ([7, 8]) where they can remain viable for decades, ensuring the persistence of genotypes produced in different years through time ([6, 9, 10]).

*Chaetoceros* is one of the most species-rich and abundant genera of planktonic diatoms with a worldwide distribution ([11]). Many species in this genus produce morphologically differentiated resting spores ([12, 13]), which are considered proxies of palaeo-productivity ([14]). Among *Chaetoceros* species*, C. socialis* often dominates phytoplankton assemblages in open oceanic and coastal waters (e.g., [15]) including the Gulf of Naples (Tyrrhenian Sea, Mediterranean Sea) where blooms are detected in spring and autumn ([16]) and its spores dominate the dormant diatom assemblage in surface sediments ([17, 18]). Recent studies have shown that the production of spores in *C. socialis* is a density-dependent process, most probably mediated by a chemical cue ([19]), and that spores act as a defense strategy against viral attacks ([20]). Similar to what was observed for other diatoms ([6]), a massive transition from vegetative cells to spores in *C. socialis* is induced by inoculating exponentially growing cells into a culture medium with low nitrogen (N) ([21]). Previous RNA-seq studies have investigated the physiological response of diatoms to N starvation ([22, 23]), a condition that negatively impacts microalgal growth in the marine environment ([24]), but none of them focused on species known to form spores.

Here, we explore gene expression profiles during spore formation induced by N starvation in *C. socialis*. After the assembly of its transcriptome, we tested the differential regulation of the major metabolic pathways, the presence of potential oxylipin-mediated signalling and other conserved pathways known to be involved in cell dormancy in other organisms. Our results provide the first account of the molecular responses during this important transition in the diatom life cycle.

## Results and discussion

**Fig. 1:**
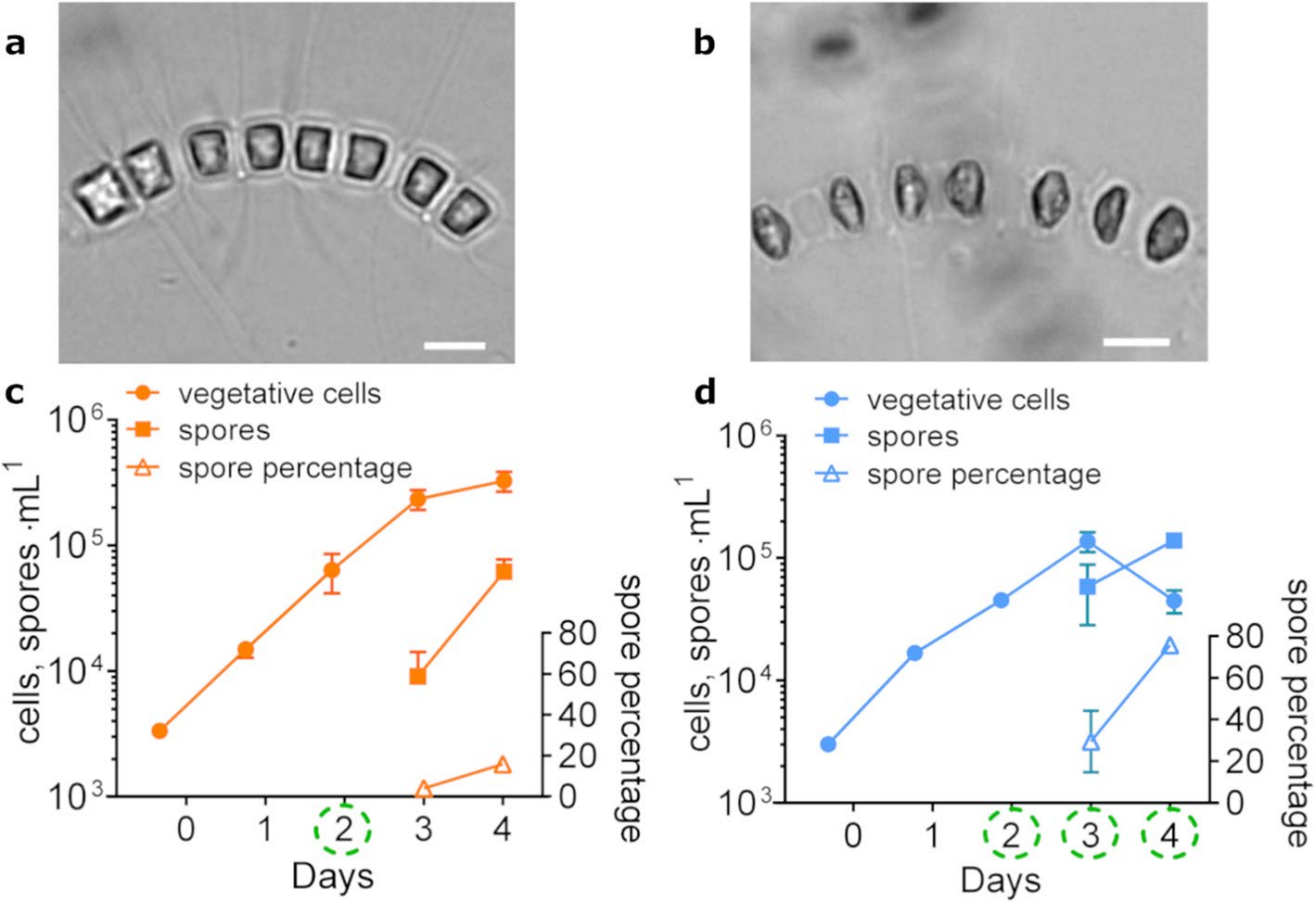
Nitrogen depletion induces the formation of resting spores in the diatom *Chaetoceros socialis*. Light micrograph of a colony of vegetative cells (a) and a colony in which vegetative cells turned into resting spores (b); scale bars = 10 µm. Time course concentration of vegetative cells and spores (cells, spores mL^−1^; left axis) and percentage of spores (right axis) in cultures grown in nitrogen-replete control (c) and in nitrogen-deplete treatment (d); data are shown as average ± S.D. (n = 3). Samples for the differential gene expression analysis were collected on day 2 in the control and on days 2 (T2), 3 (T3) and 4 (T4) for the treatment and are marked with dashed green circles in panels c and d, respectively

The efficacy of N starvation in inducing a rapid and massive transition from vegetative cells to resting spores in *C. socialis*, first shown by Pelusi et al. ([19, 21]), is here confirmed. After three days of exponential growth, *C. socialis* cells grown in N depleted medium entered in stationary phase and vegetative cells (Fig. 1a) progressively turned into spores (Fig. 1b), reaching ~ 30% and ~ 75% of the total cell number on T3 and T4, respectively (Fig. 1d). In control conditions, spores were present with a much lower percentage (~ 15%) only at T4 (Fig. 1c). The experiment was conducted with exactly the same set-up illustrated in Pelusi et al. ([19]), where concentrations of inorganic nutrients in the medium and of organic N and C in cells were monitored in both treatment and control conditions. The cell and spore concentrations over the four days of the experiment here illustrated matched the overall dynamics observed in Pelusi et al. ([19]), where spore formation in the treatment coincided with a marked increase of intracellular C (from an average value of ~ 8 pg cell^−1^ on day 0 to ~ 34 pg cell^−1^ on day 4), while the intracellular N pool remained almost constant (between ~ 1.4 pg cell^−1^ on day 0 and ~ 1.6 pg cell^−1^ on day 4). These data show that during spore formation cells keep assimilating carbon, likely to build metabolic energy reserves. The fact that the internal N pool does not decrease under N limitation in the culture medium suggests a qualitative shift in intracellular nitrogenous compounds and supports the conclusion that the internal N pool is not the primary cue for the induction of spore formation ([19]). In the transcriptomic experiment and in the experiments illustrated in [19] non-axenic cultures were used. The concentration of NO_3_ in the treatments showed a marked and constant decrease [19] and the concentration of NO_2_ and NH_4_ was always below 0.1 and 0.4 µM, respectively (A. Pelusi, unpublished data), Although the bacterial load was minimal at the sampling days chosen for this study, a possible effect of bacterial remineralization in altering inorganic nitrogen concentration and its uptake by the diatom cannot be excluded.

### Transcriptome assembly and differential expression analysis

RNA-seq experiments were performed on *C. socialis* samples collected in N deplete medium before the formation of spores (T2), when spore formation started (T3), and when spores reached > 75% of the whole population (T4) (Fig. 1d); control samples were collected from cells in mid-exponential growth phase on day 2 (C2) (Fig. 1c). Since the *C. socialis* genome is not available yet, raw reads have been assembled with a de novo approach. The assembled transcriptome accounted for a total of 32,224 transcripts (Table S1, S2), with a mean GC content of 43.98%, an average and median contig length of 776 and 536 bp, respectively, and a N50 of 1140 bp (Table S1). In silico encoded protein sequences were 19,153. Among these, 6693 (34.9%) were full-length proteins, 5401 (28.2%) were 5’ partial proteins lacking a stop codon, 2328 (12.2%) were 3’ partial proteins lacking a start methionine, and 4731 (24.7%) lacked both a starting methionine and a stop codon (Table S1). The function of 8,070 protein sequences (42.1% of the total predicted proteins) was successfully annotated (Table S2). Among these, 7,974 proteins were associated with at least one gene ontology (GO) term.

The comparisons between each time point (T2, T3 and T4) and the control (C2) were used to detect the molecular pathways involved in spore formation induced by N depletion. More than 65% of the total transcripts (21,873) resulted to be differentially expressed genes (DEGs), with a fold change log2(FC) ≥ 1.5, and the majority of them belonged to T3 and T4 (Table 1, Table S2). The hierarchical clustering of the DEGs dataset highlighted a suite of genes specifically characterizing T3 and T4, when spore formation was taking place, as compared to the control condition (Figure S1).

**Table 1:** Number of differentially expressed genes (DEGs), Gene Ontology (GO) enriched terms and pathways annotated among up- and downregulated DEGs in the different comparisons between treatments (T) and control (C)

Comparison	Up-reg DEGs	Down-reg DEGs	GO terms enriched in up-reg. DEGs	GO terms enriched in down-reg. DEGs	Pathway occurrence in up-reg. DEGs	Pathway occurrence in down-reg. DEGs
T2 vs C2	4838	388	469	142	160	14
T3 vs C2	11,704	3851	704	494	351	187
T4 vs C2	13,851	4801	699	549	429	181

### Pathway occurrence and GO enrichment

**Fig. 2:**
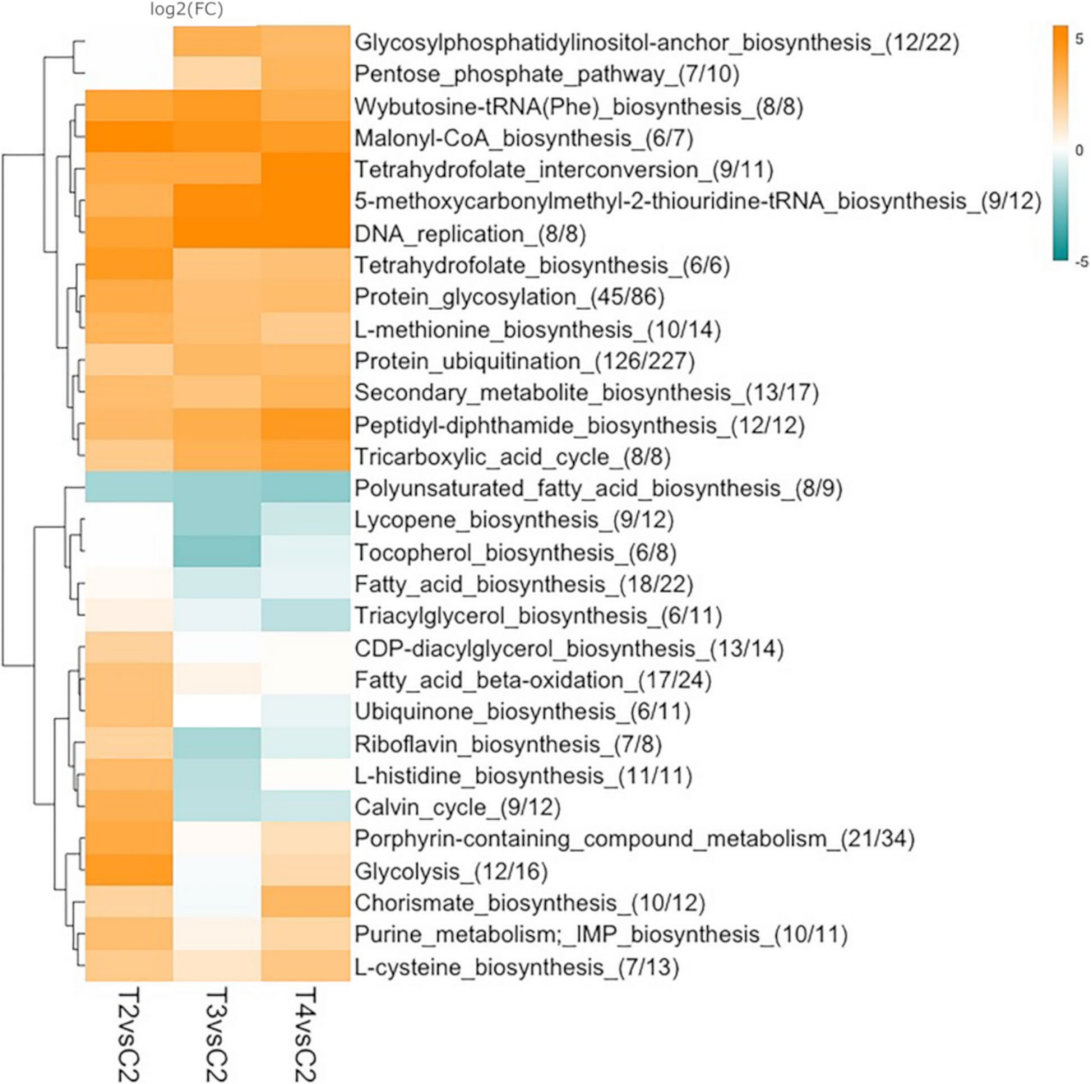
Hierarchical clustering analysis of log2(FC)s expression related to the top metabolic pathways in terms of DEGs occurrences. The heatmap represents SwissProt pathways displaying statistical significance (average log2(FC) of all the DEGs in the pathway ≥ 1.5, adjusted P-value ≤ 0.05) for differential expression between sampling points. The up-regulated pathways are reported in orange, the down-regulated pathways in blue and the non differentially expressed ones in white. For each pathway, the number of DEGs over the total number of C. *socialis* transcripts retrieving the annotation is shown in parentheses. For each condition and each pathway, the average log2(FC) of all the DEGs related to that pathway is shown with the corresponding colour

**Fig. 3:**
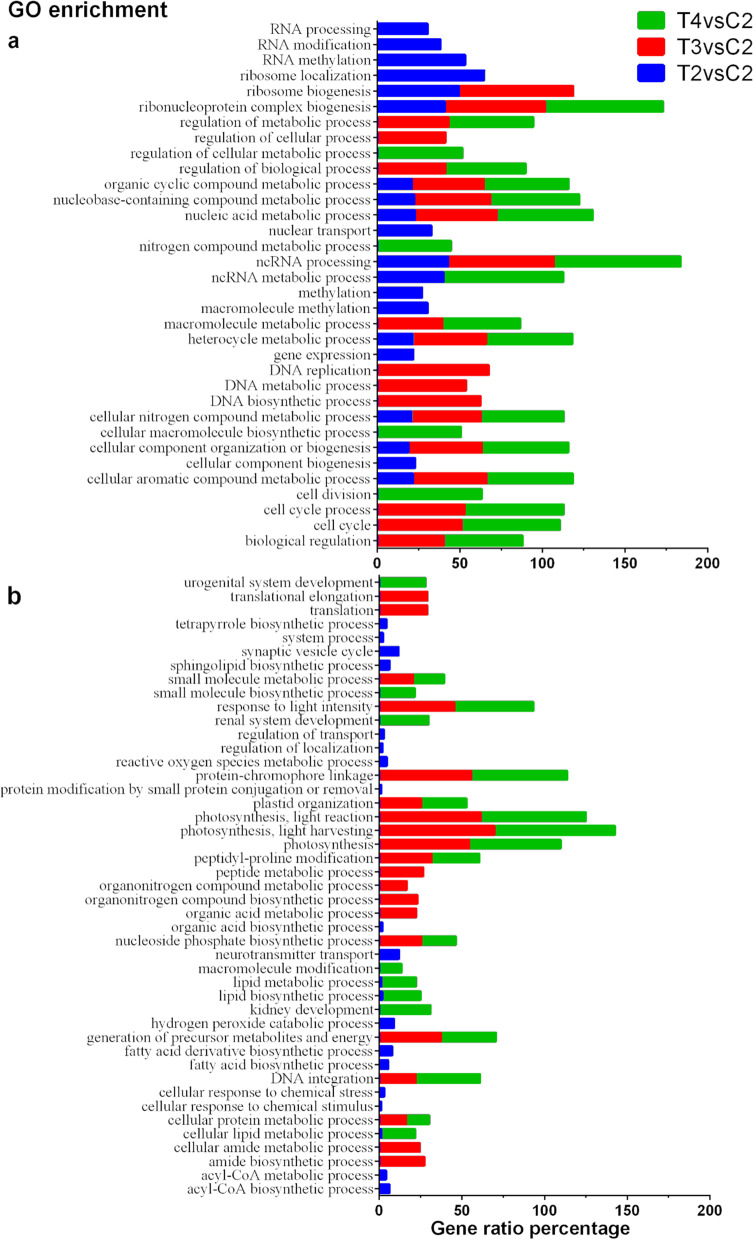
The first 20 enriched GOs terms in each comparison. Terms enriched in upregulated (a) and downregulated (b) DEGs at T2, T3 and T4 compared to control conditions are reported. The x-axis indicates gene ratio percentage, i.e., the number of genes found enriched in each comparison over all those annotated under the same term in the transcriptome

A summary of pathways with the highest DEGs occurrence and the most significantly enriched GO terms detected in each comparison is illustrated in Figs. 2 and 3, respectively. A total of 406 pathways and of 3,057 GO terms were significantly enriched in T2, T3 and T4 *versus* control condition (Table 2, Table S3 and S4). The expression profile of the pathways indicates the presence of four main clusters that highlight an overall higher similarity between T3 and T4 (Fig. 2). The same conclusion is obtained by looking at the enriched GO terms: both up- and downregulated DEGs in T2 *versus* control were very few and redundant, suggesting that the major metabolic rearrangements started at T3. In fact, the presence of spores at T3 and T4 coincides with changes in the expression of pathways and an increased number of enriched GO terms (Table 1). Among the pathways shared by all comparisons, the overexpression of *L-methionine* and *L-cysteine* biosynthesis is related to the GO terms *cellular aromatic compounds* and *cellular nitrogen compounds,* which were found positively enriched (Fig. 2 and 3a). Also the *DNA replication* and the *tetrahydrofolate biosynthesis,* related to nucleotide biosynthesis, find accordance to the GO terms *nucleobase-containing compound metabolic process* and *nucleic acids metabolic processes* over-represented at all sampling points. Specifically, an enrichment of terms relative to upregulated DEGs linked to *cell cycle* was present at T3 and T4 and to *cell division* at T4 (Fig. 3a); these observations parallel the high expression of *cyclin D3-2* (Table 2a), whose homolog in *Phaeodactylum tricornutum* was suggested to play a role in G2-to-M transition ([25]). The overexpression of these genes at first seems counterintuitive because N limitation should negatively affect cell duplication. However, this finding is supported by observations in confocal microscopy demonstrating that two consecutive acytokinetic nuclear divisions immediately precede the transformation of vegetative cells into spores ([21]). After the mitotic division, one nucleus degenerates and one new thick theca of the spore is synthesised; the process is repeated when the second theca of the spore is produced.

**Table 2:** The top 10 most over (a) and under (b) expressed genes at the different sampling points (T2, T3, T4) versus control (C2). The transcript ID, the SwissProt ID and function are reported together with the log2(FC) for the different comparisons

**a) TRANSCRIPT ID**	**SwissProt_ID**	**SwissProt_function**	**T2vsC2**	**T3vsC2**	**T4vsC2**
TRINITY_DN4348_c0_g1_i3	Q9FGQ7	Cyclin-D3-2	5.11	13.57	14.31
TRINITY_DN725_c0_g1_i4	Q53FA7	Quinone oxidoreductase PIG3	4.77	13.03	13.73
TRINITY_DN339_c0_g1_i5	Q7S045	Non-histone chromosomal protein 6	5.44	13.01	13.68
TRINITY_DN2312_c0_g1_i11	Q8BWU8	Ethanolamine-phosphate phospho-lyase	4.76	12.99	14.16
TRINITY_DN1112_c0_g1_i2	P30041	Peroxiredoxin-6	-	12.98	-
TRINITY_DN2429_c0_g1_i6	Q9NP80	Calcium-independent phospholipase A2-gamma	4.99	12.57	13.43
TRINITY_DN2494_c0_g1_i2	Q9FK72	Heat stress transcription factor A	5.54	12.56	14.01
TRINITY_DN195_c0_g1_i4	Q9FM67	Protein TIC 20-v, chloroplastic	5.89	12.46	13.81
TRINITY_DN790_c0_g1_i6	F4K2K3	ARF guanine-nucleotide exchange factor GNL2	-	12.40	12.95
TRINITY_DN1629_c0_g1_i2	P40301	Proteasome subunit alpha type-2	5.39	12.09	12.67
**b) TRANSCRIPT ID**	**SwissProt_ID**	**SwissProt_function**	**T2vsC2**	**T3vsC2**	**T4vsC2**
TRINITY_DN247_c0_g1_i1	Q9LXH0	High affinity nitrate transporter 2.6	-	-11.55	-12.20
TRINITY_DN2228_c0_g1_i3	O14283	Transcription factor prr1	-	-8.28	-4.52
TRINITY_DN22327_c0_g1_i7	B4JTF5	Protein hedgehog	-	-8.27	-6.87
TRINITY_DN7356_c0_g1_i1	Q02073	20 kDa chaperonin, chloroplastic	-	-8.26	-2.17
TRINITY_DN80_c0_g1_i2	Q7Z8P9	Nucleoside diphosphate kinase	-	-8.03	-3.52
TRINITY_DN32657_c0_g1_i1	P49534	Uncharacterized protein ycf39	-	-7.71	-3.28
TRINITY_DN20497_c0_g1_i1	Q9LYR5	Peptidyl-prolyl cis–trans isomerase, chloroplastic	-	-7.17	-3.84
TRINITY_DN4637_c0_g1_i6	O80832	UPF0187 protein, chloroplastic	-	-7.17	-3.23
TRINITY_DN22327_c0_g1_i3	Q98862	Indian hedgehog B protein	-	-7.09	-6.44
TRINITY_DN262_c0_g1_i3	Q4ING3	Cytochrome c peroxidase, mitochondrial	-	-7.02	-3.90

In analogy to what was reported for other diatoms ([23] [22]) the pathway of *protein ubiquitination,* which allows the recycling of N compounds, was upregulated at all sampling points.

Enriched GO terms among the downregulated genes in T3 and T4 compared to the control were related to photosynthesis (e.g., *photosynthesis, protein-chromophore linkage, light harvesting*), paralleled by the downregulation of the *Calvin cycle* pathway (Fig. 2 and 3b). The decline of photosynthesis during N starvation is a common response in photosynthetic organisms due to the tight connection between C and N metabolisms ([26, 27]). Cells must control their photosynthetic capability to mitigate the damage due to N starvation, since an excess of light absorption relative to C fixation can result in photo-oxidative damage that can lead to cell death. Surprisingly, the biosynthesis of tocopherol, a diatom-specific chloroplast antioxidant ([28]), was downregulated in T3 (Fig. 2). However, a number of transcripts specifically related to oxidative stress were found upregulated at T3 and T4 and will be further discussed below.

Differences in expression between T3 and T4 *versus* control of two catabolic pathways, *pentose phosphate* and *glycolysis* (with a more prominent differential expression in T4) and the anabolic *Calvin Cycle* (with a less prominent differential expression at T4), suggest differences in the metabolism between these two sampling points, i.e., from the early phase of spore formation to their maturation (Fig. 2).

### Primary metabolism during spore formation

In the following, we provide evidence derived from gene expression analysis for several metabolic pathways detected in *C. socialis* during spore formation. The 10 annotated genes with the highest up- and downregulation values in all pairwise comparisons are reported in Table 2; upregulated genes had a log2(FC) between 13 and 12 both at T3 and T4 and downregulated ones were not differentially expressed at T2, indicating their involvement during spore formation. Some of the genes were present with a high number of isoforms, such as the high-affinity urea active transporters *DUR3* and the serine/threonine-protein kinases *TOR* (see Table S6, S8), suggesting a still high level of redundancy among transcripts despite the filtering applied in the bioinformatic analysis.

In order to validate the RNA-seq dataset, we tested the expression of six genes by RT-qPCR in strain APC12 – used in the RNA-seq experiment—and in the additional, recently isolated, strain MCA6. The latter strain has been chosen to test possible variation in gene expression due to maintenance in laboratory conditions. Replicate cultures of both strains were collected when the percentage of spores was comparable to those obtained at T3 in the RNA-seq experiment (33 and 38% for APC12 and MCA6, respectively) (Figure S2a, b). All the tested genes, but *Cyclin-dependent kinase 5* homolog, showed expression trends in agreement with the results of the transcriptomic analysis (Figure ﻿S2c, d; Table S5). However, only two and four out of the six *DEGs* tested were significantly differentially expressed in APC12 and MCA6, respectively (Figure ﻿S2c, d). They included one of the most differentially expressed high-affinity nitrate transporter – *NRT 2.6*—and the silicon efflux transporter – *LSI3* – which confirmed their expression in both strains. Differences between the two strains may be attributed either to physiological variability or to the fact that APC12 was tested in RT-qPCR about three years after the RNA-seq experiment and was thus much older than MCA6.

#### Nitrogen assimilation

**Fig. 4:**
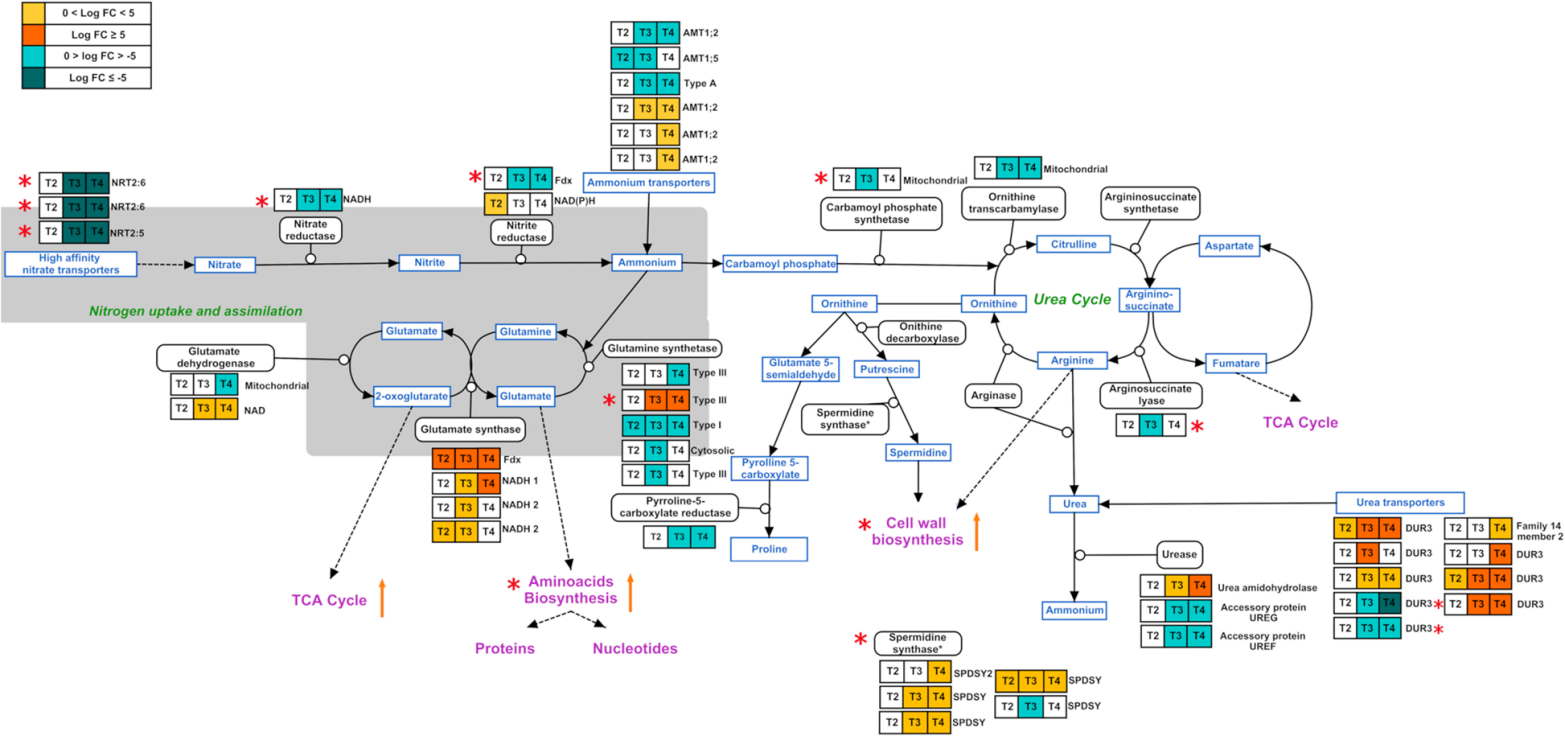
DEGs related to nitrogen metabolism during spore formation in *C. socialis* obtained by manual search. Box colors indicate the relative log2(FC) detected in the comparisons between T2 *vs* C2, T3 *vs* C2, T4 *vs* C2; orange and blue indicate up- and downregulation, respectively. The additional information obtained by SwissProt annotation is reported on the right side of each box. In the grey area are genes related to N assimilation. Red asterisks highlight differences with the expression of the non-spore former *Thalassiosira pseudonana* as in [23]; orange arrows represent upregulated pathways in *C. socialis*

Surprisingly, the high-affinity nitrate transporters (*NRTs*) in *C. socials* had a distinctive expression profile in N-starved conditions as compared to the one detected in other diatoms ([29, 30]). The assimilation of nitrate was severely impacted by N stress: three out of the four *NRTs* were in fact highly downregulated (log2(FC) between -12 and -5), both at T3 and T4, with one of them (high-affinity nitrate transporter 2.6, *NRT2.6*) being the most downregulated transcript of the whole dataset (Fig. 4, Table 2b). *NRTs* can be constitutive or inducible in microalgae, as in plants, especially in response to low N concentration ([29]). Examples among diatoms are *T. pseudonana*, *Pseudo-nitzschia multiseries* and *Phaeodactylum tricornutum*, which showed upregulation of these genes in N starvation, with the only exception of one transcript of *P. multiseries* (Pm 261,779) ([23]). In the haptophyte *Tisochrysis lutea* one of the four *NTRs* decreased in N depletion, showing an expression profile matching the decline of the intracellular N:C ratio ([31]). This ratio markedly decreased also in *C. socialis,* due to C accumulation when cells turned into spores ([19]), suggesting that a similar mechanism could explain the observed expression profile in our dataset.

We could not detect any low-affinity nitrate transporters (*NPFs*) in *C. socialis*, thus confirming the results recently obtained for various *Chaetoceros* species in which *NPFs* are missing ([33]); this could support the suggestion that the species of this genus can preferentially use ammonia, possibly from bacteria, as a source of N in case of fluctuating nitrate concentrations ([34]). While the expression of ammonium transporters was somehow variable in our dataset, the high-affinity urea active transporters *DUR3* were upregulated at least in one sampling point, with two of them already differentially expressed at T2 (Fig. 4, Figure S3a, Table S6).

Several transcripts coding for glutamine synthetase (*GS*) and glutamate synthase (*GOGAT*), key enzymes in N assimilation, have been detected (Fig. 4, Table S6). Among the *GS* transcripts, one transcript coding for *GSIII* showed a log2(FC) of 8 at T3 and T4 *versus* control, as reported for *T. pseudonana* proteome ([35]) and for the cyanobacterium *Synechococcus* ([36]) during the early phase of N starvation. Hockin et al. ([35]) suggested that in *T. pseudonana* this gene participates in the assimilation of ammonium obtained by intracellular catabolic processes, in combination with a NAD(P)H*-GOGAT*. It should be noted, however, that opposite results were obtained at transcriptional level for the same gene in *T. pseudonana* ([23]). In our dataset, only a chloroplastic ferredoxin-*GOGAT* enzyme displayed a similar positive trend at all sampling points (log2(FC) from 5 to 7), while the other enzymes using NADH as cofactor showed only slight positive regulation at T3 (Fig. 4, Table S6). It can thus be hypothesized that *C. socialis*, like other diatoms, recovers most of N by recycling the internal pools, as further suggested by the protein ubiquitination (Fig. 2) and the hydrolysis of urea by *urease*, over-expressed at T3 and T4 with log2(FC) close to 5 (Fig. 4, Figure S3a, Table S6). On the other hand, despite the upregulation of *urease,* the urea cycle transcripts did not appear differentially expressed (Fig. 4) in accordance with the observations in *T. pseudonana* ([23]). Another important source of N could be the ammonia obtained from the breakdown of phosphoethanolamines through the ethanolamine-phosphate phospho-lyase, an enzyme showing a log2(FC) > 12 when spores were detected at T3 and T4 (Figure S3a, Table S6). These different evidences of recovering N by recycling the internal pools contribute to explaining the fact that the internal N pool during spore formation in *C. socialis* remains constant ([19]).

#### Tricarboxylic acid cycle (TCA)

The TCA cycle is responsible for generating energy through the oxidation of pyruvate to form CO_2_, ATP, NADH, and carbon skeletons used for biosynthetic processes. This pathway was overexpressed during the time course of the experiment (Fig. 2). The upregulation of two isocitrate lyases with log2(FC)s of 4 and 8, respectively, only when spores were present (i.e., T3 and T4) could imply that the glyoxylate cycle, a variant of the TCA cycle that uses lipids as a source of energy, is preferred by diatom spores (Figure S3b, Table S6). The upregulation of transcripts such as the mitochondrial short-chain specific acyl-CoA dehydrogenase (log2 (FC) 2 and 6 in T3 and T4, respectively) and the peroxisomal multifunctional enzyme type 2 (*MFE-2*) that increased progressively from T2 reaching log2(FC) of 6 at T4, suggest the involvement of the beta-oxidation of fatty acids in feeding the glyoxylate cycle with acetate molecules (Figure S3c, Table S6). Indeed, a *calcium-independent phospholipase A2-gamma* responsible for membrane lipid degradation has been found among the most up-regulated genes (Table 2) with log2(FC) reaching values > 12. These metabolic changes are consistent with those detected during the formation of resting stages (pellicle cysts) in the dinoflagellate *Scripsiella trochoidea* ([37]).

#### Carbon skeletons 

Part of the carbon skeletons for sustaining the cells were obtained from the degradation of chrysolaminarin, the principal storage compound of diatoms. The exo-1,3,-beta-glucanases, presumed enzymes responsible for its degradation ([38]), were found with ten different transcripts, eight of which were upregulated particularly when spores were present with log2(FC) ranging from 1.9 to 6 (Figure ﻿﻿﻿S3d, Table S6). These skeletons most probably feed the glycolysis and the pentose phosphate pathways, both with upregulated enzymes (Fig. 2).

The pentose phosphate pathway produces NADH and pentose sugars in the oxidative and reductive phases, respectively. Among the sugars produced, the ribose 5-phosphate is the precursor of nucleotides and thus essential for DNA replication and transcription. In analogy to what was observed in N-starved *P. tricornutum* ([22]), enzymes involved in the oxidative pathway—two glucose-6-phosphate 1-dehydrogenases and two 6-phosphogluconate dehydrogenase decarboxylating 1, one chloroplastic and one cytosolic—were upregulated at T3 and T4 with log2(FC) spanning from 2 to 7 (Figure S﻿﻿3d, Table S﻿6). Transcripts of the non-oxidative part were instead generally downregulated, except for one chloroplastic transketolase, which is responsible for the production of D-xylulose 5-phosphate and D-ribose 5-phosphate and that showed a log2 (FC) increasing from 2 in T2 to 8 in T4; this latter molecule is the precursor of nucleotide biosynthesis, essential for the formation of spores.

#### Cell wall

One of the most relevant changes during the formation of spores is the deposition of two thick heteromorphic siliceous thecae that confer mechanical protection to the spores. Metabolites such as spermidine and long-chain polyamines (LCA) are involved in the synthesis of the organic component of the siliceous cell wall and their synthesis is connected to the urea cycle and the TCA cycle ([39]). Evidence of spermidine production, another polyamine, came from the upregulation of several spermidine synthases, especially when spores were present (Fig. 4, Figure S3e, Table S6). Furthermore, polyamines transporters and polyamine oxidases, involved in the regulation of their intracellular concentration, showed high log2(FC) at T3 and T4. The deposition of new silica thecae of the spores was supported also by the simultaneous increase of two silicon efflux transporters (*LSI*), with log2(FC)between 4 and 5, and of several other transcripts presenting the InterPRO domain ‘Silicon transporter’ (IPR004693) with extremely high log2(FC) (up to 11) (Figure S3e﻿, Table S6).

### Chemical signaling

#### Pathways related to oxylipin production

There are several examples of chemically mediated communication in unicellular organisms ([40]), with some of them having a critical role in dormancy ([41]). In diatoms, the most studied infochemicals related to intra- and inter-specific communication are compounds belonging to the oxylipin family [42], which include polyunsaturated aldehydes (PUAs) and linear oxygenated fatty acids (LOFAs), both derived from the oxygenation of fatty acids ([43]). The first step of their synthesis derives from the oxidation of membrane lipids, through several enzymes among which a crucial role is played by lipoxygenases. The production of LOFAs has been reported for *C. socialis,* although the enzymatic pathway involved is still unknown ([44]). A single-copy lipoxygenase with an extremely high log2(FC) (> 10) has only been detected when spores were present (i.e. T3 and T4), supporting the hypothesis of oxylipin-mediated signalling as the trigger of spore formation (Figure S4a, Table ﻿S7). The formation of spores occurs under N limitation, a stress condition that induces cell lysis and mortality, whose rate has not been possible to quantify in our study. The shift from vegetative cells to spores occurs also when *C. socialis* is attacked by viruses, which induce cell death ([20]), and when exponentially growing cultures are inoculated in culture medium obtained from sonicated vegetative cells at high concentration ([19]). All these experimental conditions share the presence of lysed/dead cells in which oxylipins can be produced following membrane breakage, corroborating the involvement of these infochemicals in determining this life cycle transition in *C. socialis*.

In addition to the overexpression of the lipoxygenase, several aldehyde dehydrogenases (*ALDHs*), plausibly linked to the detoxification from oxylipins, showed log2(FC) spanning form 2 to 13 especially at T3 and T4 (Figure S4a, Table S7﻿). These enzymes are very conserved all over the phylogenetic tree of life and have a variety of functions, spanning from detoxification against oxidative stress ([45]), to being markers for highly proliferating stem cells and cancer cell phenotypes ([46] and reference therein). We hypothesize that the simultaneous production of LOFAs and *ALDHs* in *C. socialis* can be attributed to the presence in the culture of cells undergoing different fates: LOFAs could be produced by lysed N starved cells as a response to stress, while the *ALDHs* by the fraction of cells that ‘react’ to stress starting a series of intracellular cascade signals that lead to spore formation.

#### Oxidative stress and programmed cell death (PCD)

The presence of transcripts encoding for antioxidant enzymes revealed oxidative stress during spore formation; however their expression pattern is at times contrasting. The peroxiredoxin-6 and the quinone oxidoreductase *PIG3* were among the most overexpressed genes in the dataset (Table 2a), and two chloroplastic enzymes—a glutathione synthetase and a thioredoxin-like 2–1 – had log2(FC)s ranging from 5 to 7 especially at T3 and T4 (Figure S4b, Table S﻿7). However, besides the downregulation of *tocopherol biosynthesis* pathway mentioned above, a mitochondrial cytochrome c peroxidase (Table 2) and a peroxiredoxin-2E-2 (Figure ﻿S4b, Table S﻿7) were also downregulated.

Diatoms have a surveillance system based on Ca^2+^ and nitric oxide (NO), which helps to monitor stress levels within the population, and, above a certain threshold level, cell death is induced in the population ([47, 48] [42]). Although only one out of the three nitric oxide synthases present in *C. socialis* was weakly upregulated (log2(FC) up to 2.7) in T3 and T4, several Ca^2+^-dependent protein kinases have been found with log2(FC)s ranging from 2 to 12, with the highest values detected when spores were present (Figure ﻿S4b, Table S﻿7). Evidence for PCD comes from the finding of programmed cell death proteins with log2(FC)s from 1.5 to 6, especially in T3 and T4, and from metacaspase 1 with transcripts having log2(FC) spanning from 1.7 to 7. In our experiments we only quantified cells with cytoplasmic content that could also include lysed and dying cells; attempts to quantify the rate of dead cells with Sytox green in epifluorescence light microscopy were not reliable.

The contrasting expression profile of some antioxidants transcripts and the presence of PCD-related genes suggest, once more, that within the population that are cells undergoing different fates, which hampers a precise interpretation of the biological meaning of the signals. Our interpretation is that stress conditions induced by N limitation induce oxidative stress; some cells can cope with it and survive increasing the production of antioxidants and forming resting spores, while in others PCD is induced. Similarly, a pH stress induces oxidative stress in the dinoflagellate *Peridinium gatunense* with cells either turning into resting cysts or dying via PCD ([49]).

### Genes involved in quiescence

Quiescence, i.e. a reversible state in which a cell does not divide but retains the ability to re-enter cell proliferation, presents very conserved molecular features among evolutionary distant organisms ([3]). For example, both quiescent mammalian cells and yeasts arrest the cell cycle in G1, condense chromosomes, reduce rRNA synthesis and translation and activate autophagy mechanisms becoming more resistant to different stresses ([3] and references therein). Similar features were recorded in the *C. socialis* transcriptome, as shown by the presence of genes related to autophagy, i.e., genes related to the recycle of internal compounds, together with the nuclear and cell wall rearrangement observed during spore formation. Among conserved pathways that have been related to quiescence in yeasts, there are several serine/threonine-protein kinases *TOR*; the activity of this enzyme decreases in N starved *Saccharomyces cerevisiae*, inducing the formation of spores ([50]). Different transcripts related to *TOR* showed a marked positive expression (log2(FC)s from 3.5 to 6) when spores were present (T3 and T4) (Figure S5, Table S8). However, the same trend was observed for its inhibitor *GATOR* supporting once more the fact that different signals are produced by cells undergoing different fates, i.e. dying cells and cells that turn into spores.

Recent studies on resting cells in the dinoflagellate *Scrippsiella trochoidea* and the diatom *P. tricornutum* reported the possible involvement of the phytohormone abscisic acid (ABA) in regulating the transition between active cell division and quiescence ([37, 51, 52]). This molecule is a well-known signal initiator in seed dormancy but it is still poorly studied in microalgae ([37]). In *C. socialis*, the expression of 9-cis-epoxycarotenoid dioxygenase, the rate-limiting enzyme of the ABA biosynthetic pathway, increased concomitantly to spore production (log2(FC)s from 2.6 to 6 in T3 and T4 *versus* control comparisons); the farnesyltransferase subunit beta is an essential part of the farnesyltransferase complex, an ABA negative regulator and maintained high log2(FC)s (from 5.3 to 6.7) at the same sampling points (Figure ﻿S5, Table S8﻿).

### Comparison with *Thalassiosira pseudonana*

**Fig. 5:**
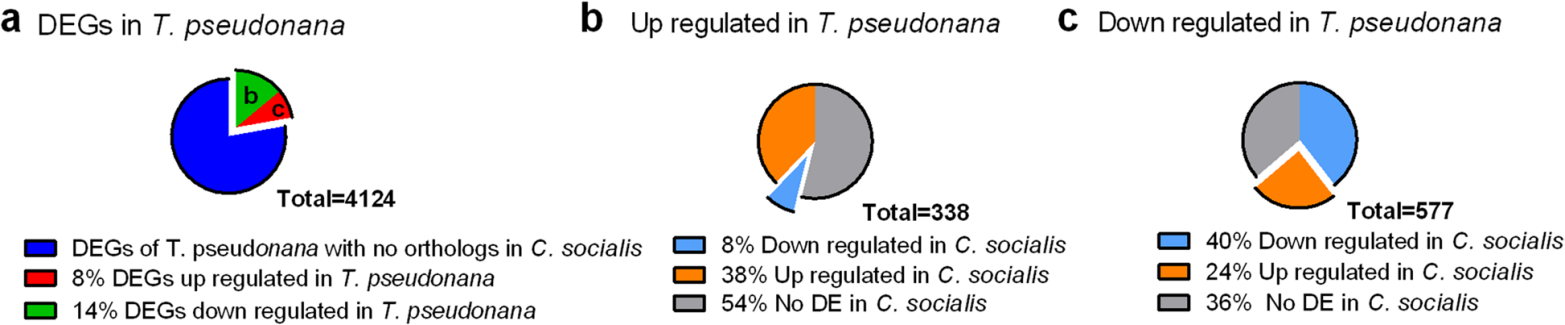
Comparison between the transcriptome of *T. pseudonana* (non- spores forming) and *C. socialis* (spore former) during the early phase of N limitation*.* DEGs of *T. pseudonana* with an ortholog counterpart in *C. socialis* (a); expression trends of the up- (b) and downregulated (c) genes, i.e., the ‘b’ and ‘c’ fractions in panel a

To gain insights into the molecular mechanisms involved in spore formation, we compared our dataset to the one produced for *T. pseudonana*, a centric diatom that does not produce resting spores and for which a similar experimental set-up was used ([23]). Bender et al. ([23]) compared the expression profile of *T. pseudonana* at the onset of the stationary phase in N-limiting conditions to the one obtained in the mid-exponential phase in nutrient replete conditions; these sampling points correspond to the beginning of spore formation in T3 and the control collected on day 2, respectively. A total of 338 upregulated and 577 downregulated genes (out of 4124 DEGs) in *T. pseudonana* had an ortholog counterpart in the spore former *C. socialis* (Fig. 5a), but only 157 and 120 genes, respectively, had an annotation in *C. socialis* (Table S9); about 40% of these orthologs showed the same trend in both species(Fig. 5 b, c).

Among the upregulated transcripts of *T. pseudonana* with the same trend in *C. socialis*, the log2(FCs) ranged from 2 to 11 and genes were linked to ribosome biogenesis, regulation of transcription and regulation of cell cycle. Orthologs sharing common downregulation, with log2(FC) spanning from -8 to -1.5, were related to fatty acids, tocopherol and amino acid biosynthesis, together with genes related to the chloroplast components and photosynthesis such as the ribulose-1,5-bisphosphate carboxylase/oxygenase large subunit N-methyltransferase (RuBisCO methyltransferase) (Table S9). These data generally confirm the metabolic response obtained with the pathway analysis and GO enrichment analysis in *C. socialis*.

No information could be gained from the analysis of upregulated transcripts showing an opposite trend in *C. socialis* (Figure S﻿﻿6a) due to the scanty annotation and weak induction (log2(FC) ≥ -3) in the latter species. The degree of expression of the downregulated genes of *T. pseudonana* upregulated in *C. socialis* (Figure S﻿6b) showed log2(FC) spanning from 2 to 15, and included genes related to mitotic division, DNA replication and fatty acid oxidation (Table S9﻿). This result supports the fact that in *T. pseudonana* there is no evidence for the mitotic cell division that accompanied spore formation in *C. socialis.* Noteworthy is the fact of the several DEGs related to N metabolism manually found in *C. socialis* (Fig. 4), only two transcripts, the urease accessory protein F and the mitochondrial ornithine transcarbamylase, had an ortholog counterpart in *T. pseudonana* presenting the same trend in both species.

These observations suggest that the comparison between the two transcriptomes has to be taken with caution due to the high redundancy of transcripts in our dataset that could be reduced when the *C. socialis* genome becomes available.

### Conclusion

The results of our study provide a first insight into the metabolic pathways activated in the centric diatom *C. socialis* during the transition from vegetative growth to the formation of resting spores. Genes related to photosynthesis and nitrate assimilation were down-regulated when spores were produced and the concomitant upregulation of a glutamine synthetase (*GS*), *ureases*,* ethanolamine-phosphate phospho-lyase* suggests that N is recovered by recycling internal pools. The upregulation of spermidine synthases and polyamines transporters, together with silicon transporters and the enrichment of terms related to cell division provide functional evidence for the active synthesis of the spores’ siliceous walls. Transcriptome data also provided evidence for signaling cues and mechanisms possibly involved in the formation of *Chaetoceros* spores. A highly overexpressed *lipoxygenase* together with several *aldehyde dehydrogenases* were detected during spore formation, suggesting the involvement of oxylipins as chemical cues. The detection of different transcripts related to the serine/threonine-protein kinase *TOR* together with its inhibitor (*GATOR*) suggests their possible involvement in the formation of diatom spores, in analogy to their role in the induction of quiescence in yeast.

The expression profile of a number of genes was in apparent contrast, i.e., the overexpression of transcripts related to cell division and PCD, or the contrasting expression profile of some antioxidants transcripts. The complex molecular signals detected in our dataset can be explained by the simultaneous presence at T3 and T4 of distinct cell types undergoing different fates. Also within a genetically identical population of unicellular organisms, physiologically and phenotypically different cells often co-exist [53]. Examples are cells with different susceptibility to oxidative stress [54], the differentiation of morphologically and functionally different life stages in budding yeast following nutrient deprivation [55], or even the differentiation of gametes during the sexual phase of diatoms [56]. We hypothesize that in *C. socialis,* vegetative cells negatively impacted by N starvation produce chemical signals that determine the transformation into resting stages of a fraction of the population. As a consequence, contrasting genetic programs are detected in the transcriptomic dataset.

In our experiments the formation of spores was induced by N depletion and we present an overview of the metabolic response that takes place under this specific experimental condition. This set up allowed to identify only a limited number of pathways putatively related to spore formation but provides the first transcriptomic dataset that can be used in further comparative experiments testing the formation of spores induced by other biological cues (e.g., ([19, 20]). Detailed investigations at the level of individual cells, taking advantage of recently established protocols, which allow the isolation of the nuclei ([57]) and the consequent possibility to carry out RNA-seq approaches on the distinct sub-populations, together with the availability of the genome of *C. socialis*, will hopefully enable to advance our understanding of the molecular mechanisms that regulate this important life cycle transition.

## Materials and methods

### Experimental set-up, RNA extraction and sequencing

The experiment was carried out using a newly established clonal strain of *C*. *socialis,* APC12, genotyped by sequencing the LSU rDNA region ([21]). A non-axenic stock has been maintained in a culture chamber at 18 ± 2 °C, under sinusoidal illumination (12L:12D h photoperiod, ~ 90 μmol photons·m^−2^· s^−1^ daily average) in control medium made with artificial seawater at a salinity of 36 (Sea salts, Sigma-Aldrich; [21]) and with the following concentration of inorganic nutrients: 580 μM of NaNO_3_, 300 μM of Na_2_SiO_3_ and 29 μM of NaH_2_PO_4_. An exponentially growing culture was used to inoculate, at an initial cell density of ~ 3 × 10^3^ cells·mL^−1^, three 5 L glass flasks filled with 3 L of control medium and three flasks filled with low nitrate medium (23 μM of NaNO_3_, 300 μM Na_2_SiO_3_, 29 μM NaH_2_PO_4_). Temperature and light conditions were monitored during the experiment with a HOBO Pendant® Temperature/Light Data Logger. To estimate cell concentration, 4 mL of sample were collected every day, fixed with 1.6% formaldehyde solution, and vegetative cells and spores were enumerated using a Sedgwick-Rafter chamber on a Zeiss Axiophot (ZEISS, Oberkochen, Germany) microscope at 400 × magnification.

**Fig. 6:**
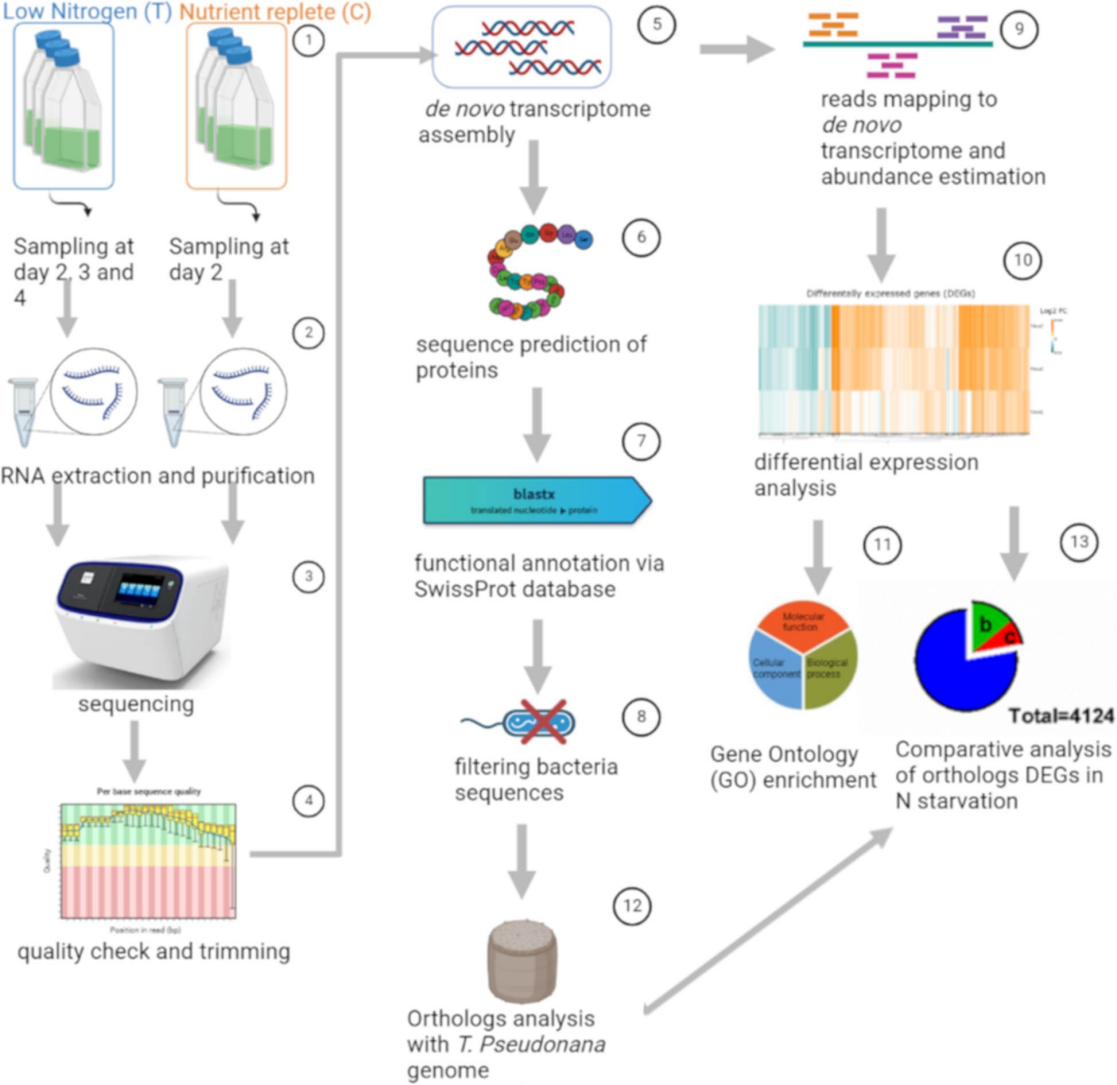
Schematic representation of the bioinformatic pipeline used in this study

Total RNA was extracted from each replicate of the control in mid-exponential growth phase at day 2 (C2) and from the replicates growing in N deplete conditions on three consecutive days: before the formation of spores (T2), when spore formation started (T3), and when they reached > 75% of the whole population (T4) (Fig. 6). A total of ~ 1.2 × 10^7^ cells were harvested from each replicate by filtration onto 1.2 μm pore size filters (RAWP04700 Millipore) and extracted with Trizol™ (Invitrogen) following manufacturer’s instructions. A DNase I (Qiagen) treatment was applied to remove gDNA contamination, and RNA was further purified using RNeasy Plant Mini Kit (Qiagen). All samples were quantified with Qubit® 2.0 Fluorometer (Invitrogen) and quality checked with an Agilent 2100 bioanalyzer (Agilent Technologies, California, USA) and a NanoDrop ND-1000 Spectrophotometer (Nanodrop Tecnologies Inc., Wilmington, USA). Samples were then pooled in equal concentrations of 100 ng·μl^−1^ for sequencing at the Molecular Service of Stazione Zoologica with an Ion Proton™ sequencer (Life Technologies, Carlsbad, USA) using an Ion P1 sequencing Kit v2, generating single-read sequences. Highly abundant ribosomal RNAs (rRNA) were removed from total RNA by positive polyA + selection. Raw reads coming from each replicate were collected in fastqc format files. One of the T3 replicates was removed from downstream analyses due to a sequencing error during library construction. The resulting raw reads were deposited in the Sequence Read Archive (SRA) partition at NCBI with the accession number PRJNA826817.

### Reads quality check, transcriptome assembly and functional annotation

The reads quality check was performed using FASTQC ([58]). A trimming step of the low-quality bases at 5’ and 3’ was performed using Trimmomatic ([59]). Low-quality nucleotides were trimmed from the ends of the reads (first 8 bases), setting the minimum quality per base at a Phread score of 20 and minimum and maximum length of the reads after cleaning at 25 bp and 240 bp, respectively. Cleaned reads were assembled into transcript sequences using Trinity v.2.11.0 ([60]) with in silico read normalization, setting the -min_kmer_cov parameter at 2. The clustering of the transcriptome was performed using the CD-hit-est software (v. 4.6.8, [61],) with 90% identity threshold in order to remove transcriptome redundancy. The whole transcriptome was aligned with BLASTx software ([62]) versus the Uniprot SwissProt database (downloaded in July 2020), setting the e-value threshold to 1e^−3^. A filtering step was performed at this stage for removing all the matches against bacterial sequences from the transcriptome.

The prediction of the encoded proteins from the assembled transcripts was obtained via TransDecoder v 5.3.0 (https://github.com/TransDecoder/TransDecoder/releases). Coding sequences were identified by the software based on: 1) a minimum length Open Reading Frame (100 by default to minimize the number of false positives); 2) an internal score system; 3) if a candidate ORF is entirely included within the coordinates of another candidate ORF, the longer one is reported. The functional annotation of the predicted proteins was performed by InterProScan (version 5.33) ([63]).

### Differentially Expressed Genes (DEGs) calling, gene ontology enrichment and comparative analysis

All the cleaned reads were mapped on the assembled *C. socialis* transcriptome using the Bowtie2 aligner (default settings, [64]). Reads count and FPKM calculation per tag for each replicate was performed using the eXpress software ([65]). DEGs calling was performed using two tools implementing two different statistical approaches: DESeq2 ([66]) and edgeR ([67]). The mean of the log2 FC values (log2(FC)) obtained with the two tools was calculated for each transcript. The thresholds for the DEGs calling were FDR ≤ 0.05, P-adjusted ≤ 0.05, and log2(FC) ≥ 1.5|. The union of the DEGs detected by both programs was retained.

A Gene Ontology enrichment analysis of the detected DEGs was performed with Ontologizer software ([68]). The threshold used to identify significantly enriched functional terms was P ≤ 0.05. Genes known to be related to different metabolic pathways were manually searched within the transcriptome considering their SwissProt annotation.

A comparative analysis was performed between the transcriptomes of *C. socialis* and *T. pseudonana,* which was also studied in nitrogen-limited experimental conditions ([23]) in non-axenic conditions. The prediction of orthologs was carried out by COMPARO, an in-house software written in python programming language ([69]).

### Real-time qPCR

Six DEGs were validated through a real-time qPCR analysis (Table S5). Three DEGs were randomly chosen, in addition to the most downregulated high-affinity nitrate transporter (*NTR2:6*) and one NADH-nitrate reductase, which are related to nitrate uptake, and a silicon efflux transporter (*LSI3*) related to the deposition of silicon in spore valves. Two genotyped strains of *C. socialis*, namely APC12 and MCA6 were used for this purpose: the former strain is the one used for the transcriptome experiment, while MCA6 is a freshly established strain isolated at station LTER-MC in the Gulf of Naples and for which the D1–D3 region of the nuclear-encoded large subunit ribosomal DNA (partial 28S rDNA) has been sequenced as in [70] to confirm its identity.

Triplicate cultures of both strains were maintained in control and low N media, with the same nutrient concentrations used for the RNA-seq experiment. Cells were harvested on day 2 in the control, when the percentage of spores was zero, and on day 3 in the treatments, when the percentage of spores was ~ 33 and ~ 38% for APC12 and MCA6, respectively, corresponding to the ones recorded at T3 of the transcriptome experiment. RNA extraction and purification were performed as illustrated above. Total RNA was reverse-transcribed using the QuantiTect® Reverse Transcription Kit (Qiagen, Venlo, Limburgo, Nederlands).

RTqPCR amplification was performed with cDNA diluted 1:10, in a 10 µl reaction containing each primer at a final concentration of 1 µM and Fast SYBR Green Master mix with ROX (Applied Biosystems) using a ViiA™ 7 Real-Time PCR System (Applied Biosystems by Life Technologies, Carlsbad, CA, USA) and the following cycling parameters: 95 °C for 20 s, 40 cycles at 95 °C for 1 s, 60 °C for 20 s, 95 °C for 15 s, 60 °C 1 min, and a gradient from 60 °C to 95 °C for 15 min. Raw results were processed using the ViiA™ 7 Software and exported into Microsoft Excel for further analyses. The reference gene used was the tubulin gamma chain (*TUB* G) designed using sequence information from the transcriptome and the software Primer3Plus v.2.4.2 ([71]). The sequences for the forward and reverse primers are 5’- TGCAGAGTTTGGTCGATGAG -3’and 5’-GGAAGCCAAAGAGTCTGCTG-3’, respectively, yielding a PCR product of 197 bp (Table S5). Primers for all other tested DEGs were designed using the same approach. log2(FC)s were obtained with the Relative Expression Software Tool-Multiple Condition Solver (REST-MCS) ([72]). A pairwise fixed reallocation randomisation test has been used to identify statistically significant results (*P* ≤ 0.05).

## Supplementary Information

**Additional file 1.** Figure S1

**Additional file 2.** Figure S2

**Additional file 3.** Figure S3

**Additional file 4.** Figure S4

**Additional file 5.** Figure S5

**Additional file 6.** Figure S6

**Additional file 7.** Table S1

**Additional file 8**. Table S2

**Additional file 9.** Table S3

**Additional file 10**. Table S4

**Additional file 11.** Table S5

**Additional file 12.** Table S6

**Additional file 13.** Table S7

**Additional file 14.** Table S8

**Additional file 15.** Table S9

## Data Availability

The dataset generated during the current study is available in the Archive Sequence Read (SRA) partition at NCBI repository deposited with the accession number PRJNA826817. All data analysed during this study are included in this published article and its supplementary information files.

## Abbreviations

NTR: High-affinity nitrate transporter
ALDHs: Aldehyde dehydrogenases
GO: Gene ontology
DEGs: Differentially expressed genes
FC: Fold change
NPFs: Low-affinity nitrate transporters
DUR3: Urea active transporters
GS: Glutamine synthetase
GOGAT: Glutamate synthase
TCA: Tricarboxylic acid
MFE: Multifunctional enzyme type
LCA: Long-chain polyamines
LSIs: Silicon efflux transporters
PUAs: Polyunsaturated aldehydes
LOFAs: Linear oxygenated fatty acids
PCD: Programmed cell death
NO: Nitric oxide
TORC1: Target of rapamycin complex I
ABA: Abscisic acid
RTqPCR: Real-time quantitative polymerase chain reaction

## References

[1] Lennon JT, Jones SE (2011). Microbial seed banks: the ecological and evolutionary implications of dormancy. Nat Rev Microbiol.

[2] Ellegaard M, Ribeiro S (2018). The long-term persistence of phytoplankton resting stages in aquatic 'seed banks'. Biol Rev.

[3] Valcourt JR, Lemons JMS, Haley EM, Kojima M, Demuren OO, Coller HA (2012). Staying alive Metabolic adaptations to quiescence. Cell Cycle.

[4] Treguer P, Bowler C, Moriceau B, Dutkiewicz S, Gehlen M, Aumont O (2018). Influence of diatom diversity on the ocean biological carbon pump. Nat Geosci.

[5] Smetacek V (1999). Diatoms and the ocean carbon cycle. Protist.

[6] McQuoid MR (1996). Diatom resting stages. J Phycol.

[7] Rynearson TA, Richardson K, Lampitt RS, Sieracki ME, Poulton AJ, Lyngsgaard MM (2013). Major contribution of diatom resting spores to vertical flux in the sub-polar North Atlantic. Deep-Sea Res Pt.

[8] Rembauville M, Manno C, Tarling GA, Blain S, Salter I (2016). Strong contribution of diatom resting spores to deep-sea carbon transfer in naturally iron-fertilized waters downstream of South Georgia. Deep-Sea Res Pt.

[9] Sicko-Goad L, Stoermer E, Kociolek J (1989). Diatom resting cell rejuvenation and formation: time course, species records and distribution. J Plankton Res.

[10] Härnström K, Ellegaard M, Andersen TJ, Godhe A (2011). Hundred years of genetic structure in a sediment revived diatom population. P Natl Acad Sci USA.

[11] Malviya S, Scalco E, Audic S, Vincenta F, Veluchamy A, Poulain J (2016). Insights into global diatom distribution and diversity in the world's ocean. P Natl Acad Sci USA.

[12] Ishii KI, Iwataki M, Matsuoka K, Imai I (2011). Proposal of identification criteria for resting spores of *Chaetoceros* species (Bacillariophyceae) from a temperate coastal sea. Phycologia.

[13] Belmonte G, Rubino F (2019). Resting cysts from coastal marine plankton. Oceanogr Mar Biol.

[14] Abelmann A, Gersonde R, Cortese G, Kuhn G, Smetacek V. Extensive phytoplankton blooms in the Atlantic sector of the glacial Southern Ocean. Paleoceanography. 2006;21(1):PA1013.

[15] Leblanc K, Aristegui J, Armand L, Assmy P, Beker B, Bode A (2012). A global diatom database - abundance, biovolume and biomass in the world ocean. Earth Syst Sci Data.

[16] Zingone A, D'Alelio D, Mazzocchi MG, Montresor M, Sarno D, Balestra C (2019). Time series and beyond: multifaceted plankton research at a marine Mediterranean LTER site. Nat Conserv.

[17] Montresor M, Di Prisco C, Sarno D, Margiotta F, Zingone A (2013). Diversity and germination patterns of diatom resting stages at a coastal Mediterranean site. Mar Ecol-Prog Ser.

[18] Piredda R, Sarno D, Lange CB, Tomasino MP, Zingone A, Montresor M (2017). Diatom resting stages in surface sediments: a pilot study comparing Next Generation Sequencing and Serial Dilution Cultures. Cryptogamie Algol.

[19] Pelusi A, Margiotta F, Passarelli A, Ferrante MI, Ribera d'Alcala M, Montresor M (2020). Density-dependent mechanisms regulate spore formation in the diatom *Chaetoceros socialis*. Limnol Oceanogr Letters.

[20] Pelusi A, De Luca P, Manfellotto F, Thamatrakoln K, Bidle KD, Montresor M (2021). Virus-induced spore formation as a defense mechanism in marine diatoms. New Phytol.

[21] Pelusi A, Santelia ME, Benvenuto G, Godhe A, Montresor M (2020). The diatom *Chaetoceros socialis*: spore formation and preservation. Eur J Phycol.

[22] Alipanah L, Rohloff J, Winge P, Bones AM, Brembu T (2015). Whole-cell response to nitrogen deprivation in the diatom *Phaeodactylum tricornutum*. J Exp Bot.

[23] Bender SJ, Durkin CA, Berthiaume CT, Morales RL, Armbrust E (2014). Transcriptional responses of three model diatoms to nitrate limitation of growth. Front Mar Sci.

[24] Berges JA, Falkowski PG (1998). Physiological stress and cell death in marine phytoplankton: induction of proteases in response to nitrogen or light limitation. Limnol Oceanogr.

[25] Huysman MJ, Martens C, Vandepoele K, Gillard J, Rayko E, Heijde M (2010). Genome-wide analysis of the diatom cell cycle unveils a novel type of cyclins involved in environmental signaling. Genome Biol.

[26] Cullen JJ, Yang XL, Falkowski PG, Woodhead AD, Vivirito K (1992). Macintyre HL Nutrient limitation of marine photosynthesis. primary productivity and biogeochemical cycles in the sea.

[27] Liefer JD, Garg A, Campbell DA, Irwin AJ, Finkel ZV (2018). Nitrogen starvation induces distinct photosynthetic responses and recovery dynamics in diatoms and prasinophytes. PLoS ONE.

[28] Nonoyama T, Kazamia E, Nawaly H, Gao X, Tsuji Y, Matsuda Y, et al. Metabolic innovations underpinning the origin and diversification of the diatom chloroplast. Biomolecules. 2019;9(8). 10.3390/biom9080322 PMC6723447 31366180

[29] Rogato A, Amato A, Iudicone D, Chiurazzi M, Ferrante MI, Ribera DM (2015). The diatom molecular toolkit to handle nitrogen uptake. Mar Genom.

[30] Busseni G, Vieira FRJ, Amato A, Pelletier E, Karlusich JJP, Ferrante MI (2019). Meta-omics reveals genetic flexibility of diatom nitrogen transporters in response to environmental changes. Mol Biol Evol.

[31] Charrier A, Berard JB, Bougaran G, Carrier G, Lukomska E, Schreiber N (2015). High-affinity nitrate/nitrite transporter genes (Nrt2) in Tisochrysis lutea: identification and expression analyses reveal some interesting specificities of Haptophyta microalgae. Physiol Plantarum.

[32] Vidal EA, Alvarez JM, Araus V, Riveras E, Brooks MD, Krouk G (2020). Nitrate in 2020: thirty years from transport to signaling networks. Plant Cell.

[33] Santin A, Caputi L, Longo A, Chiurazzi M, Ribera d'Alcala M, Russo MT (2021). Integrative omics identification, evolutionary and structural analysis of low affinity nitrate transporters in diatoms, diNPFs. Open Biol.

[34] Olofsson M, Robertson EK, Edler L, Arneborg L, Whitehouse MJ, Ploug H (2019). Nitrate and ammonium fluxes to diatoms and dinoflagellates at a single cell level in mixed field communities in the sea. Sci Rep.

[35] Hockin NL, Mock T, Mulholland F, Kopriva S, Malin G (2012). The response of diatom central carbon metabolism to nitrogen starvation is different from that of green algae and higher plants. Plant Physiol.

[36] Sauer J, Dirmeier U, Forchhammer K (2000). The *Synechococcus* strain PCC 7942 glnN product (glutamine synthetase III) helps recovery from prolonged nitrogen chlorosis. J Bacteriol.

[37] Guo X, Wang ZH, Liu L, Li Y (2021). Transcriptome and metabolome analyses of cold and darkness-induced pellicle cysts of *Scrippsiella trochoidea*. BMC Genomics.

[38] Kroth PG, Chiovitti A, Gruber A, Martin-Jezequel V, Mock T, Parker MS (2008). A model for carbohydrate metabolism in the diatom *Phaeodactylum tricornutum* deduced from comparative whole genome analysis. PLoS ONE.

[39] Prihoda J, Tanaka A, de Paula WBM, Allen JF, Tirichine L, Bowler C (2012). Chloroplast-mitochondria cross-talk in diatoms. J Exp Bot.

[40] Venuleo M, Raven JA, Giordano M (2017). Intraspecific chemical communication in microalgae. New Phytol.

[41] Waters CM, Bassler BL (2005). Quorum sensing: cell-to-cell communication in bacteria. Annu Rev Cell Dev Biol.

[42] Graff van Creveld S, Mizrachi A, Vardi A. An ocean of signals: intracellular and extracellular signaling in diatoms. In: Falciatore A, Mock T, editors. The molecular life of diatoms. Springer; 2022. p.641–678

[43] Orefice I, Di Dato V, Sardo A, Lauritano C, Romano G (2022). Lipid mediators in marine diatoms. Aquatic Ecol.

[44] Fontana A, d'Ippolito G, Cutignano A, Romano G, Lamari N, Gallucci A (2007). LOX-induced lipid peroxidation mechanism responsible for the detrimental effect of marine diatoms on zooplankton grazers. ChemBioChem.

[45] Kotchoni SO, Jimenez-Lopez JC, Kayode APP, Gachomo EW, Baba-Moussa L (2012). The soybean aldehyde dehydrogenase (ALDH) protein superfamily. Gene.

[46] Hegab AE, Ha VL, Bisht B, Darmawan DO, Ooi AT, Zhang KX (2014). Aldehyde dehydrogenase activity enriches for proximal airway basal stem cells and promotes their proliferation. Stem Cells Dev.

[47] Vardi A, Formiggini F, Casotti R, De Martino A, Ribalet F, Miralto A (2006). A stress surveillance system based on calcium and nitric oxide in marine diatoms. Plos Biol.

[48] Vardi A, Bidie KD, Kwityn C, Hirsh DJ, Thompson SM, Callow JA (2008). A diatom gene regulating nitric-oxide signaling and susceptibility to diatom-derived aldehydes. Current Biol.

[49] Vardi A, Berman-Frank I, Rozenberg T, Hadas O, Kaplan A, Levine A (1999). Programmed cell death of the dinoflagellate *Peridinium gatunense* is mediated by CO(2) limitation and oxidative stress. Curr Biol.

[50] Sun SY, Gresham D (2021). Cellular quiescence in budding yeast. Yeast.

[51] Liu XH, Wang LJ, Wu SC, Zhou L, Gao S, Xie XJ (2022). Formation of resting cells is accompanied with enrichment of ferritin in marine diatom *Phaeodactylum tricornutum*. Algal Res.

[52] Deng YY, Hu ZX, Shang LX, Peng QC, Tang YZ (2017). Transcriptomic analyses of *Scrippsiella trochoidea* reveals processes regulating encystment and dormancy in the life cycle of a dinoflagellate, with a particular attention to the role of abscisic acid. Front Microbiol.

[53] Ackermann M (2015). A functional perspective on phenotypic heterogeneity in microorganisms. Nat Rev Microbiol.

[54] Mizrachi A, van Creveld SG, Shapiro OH, Rosenwasser S, Vardi A. Light-dependent single-cell heterogeneity in the chloroplast redox state regulates cell fate in a marine diatom. Elife. 2019;8. 10.7554/eLife.47732 PMC6682412 31232691

[55] Honigberg SM (2016). Similar environments but diverse fates: Responses of budding yeast to nutrient deprivation. Microbial Cell.

[56] Bilcke G, Immacolata Ferrante M, Montresor M, De Decker S, De Veylder L, Vyverman W, Falciatore A, Mock T (2022). Life cycle regulation. The molecular life of diatoms.

[57] Annunziata R, Balestra C, Marotta P, Ruggiero A, Manfellotto F, Benvenuto G (2021). An optimised method for intact nuclei isolation from diatoms. Sci Rep.

[58] Andrews S. Babraham bioinformatics-FastQC a quality control tool for high throughput sequence data. URL:. 2010.

[59] Bolger AM, Lohse M, Usadel B (2014). Trimmomatic: a flexible trimmer for Illumina sequence data. Bioinformatics.

[60] Grabherr MG, Haas BJ, Yassour M, Levin JZ, Thompson DA, Amit I (2011). Trinity: reconstructing a full-length transcriptome without a genome from RNA-Seq data. Nature Biotechnol.

[61] Li W, Godzik A (2006). Cd-hit: a fast program for clustering and comparing large sets of protein or nucleotide sequences. Bioinformatics.

[62] Camacho C, Coulouris G, Avagyan V, Ma N, Papadopoulos J, Bealer K (2009). BLAST+: architecture and applications. BMC Bioinformatics.

[63] Jones P, Binns D, Chang H-Y, Fraser M, Li W, McAnulla C (2014). InterProScan 5: genome-scale protein function classification. Bioinformatics.

[64] Langmead B, Salzberg SL (2012). Fast gapped-read alignment with Bowtie 2. Nat Methods.

[65] Roberts A, Trapnell C, Donaghey J, Rinn JL, Pachter L (2011). Improving RNA-Seq expression estimates by correcting for fragment bias. Genome Biol.

[66] Love MI, Huber W, Anders S (2014). Moderated estimation of fold change and dispersion for RNA-seq data with DESeq2. Genome Biol.

[67] Robinson MD, McCarthy DJ, Smyth GK (2010). edgeR: a Bioconductor package for differential expression analysis of digital gene expression data. Bioinformatics.

[68] Bauer S, Grossmann S, Vingron M, Robinson PN. Ontologizer 2.0—a multifunctional tool for GO term enrichment analysis and data exploration. Bioinformatics. 2008;24(14):1650–1. 10.1093/bioinformatics/btn250 18511468

[69] Ambrosino L, Ruggieri V, Bostan H, Miralto M, Vitulo N, Zouine M (2018). Multilevel comparative bioinformatics to investigate evolutionary relationships and specificities in gene annotations: an example for tomato and grapevine. BMC Bioinformatics.

[70] Gaonkar CC, Kooistra W, Lange CB, Montresor M, Sarno D. Two new species in the *Chaetoceros socialis* complex (Bacillariophyta): *C. sporotruncatus* and *C. dichatoensis*, and characterization of its relatives, *C. radicans* and *C. cinctus*. J Phycol. 2017;53(4):889–907. 10.1111/jpy.12554 28593733

[71] Untergasser A, Cutcutache I, Koressaar T, Ye J, Faircloth BC, Remm M (2012). Primer3-new capabilities and interfaces. Nucleic Acids Res.

[72] Pfaffl MW, Horgan GW, Dempfle L (2002). Relative expression software tool (REST (c)) for group-wise comparison and statistical analysis of relative expression results in real-time PCR. Nucleic Acids Res.

