# Supplementary material for: Gene expression during the formation of resting spores induced by nitrogen starvation in the marine diatom Chaetoceros socialis
Source: BMC Genomics. 2023 Mar 10;24:106. doi: 10.1186/s12864-023-09175-x (PMC9999646; doi:10.1186/s12864-023-09175-x)
Supplement: Supplementary file 1 — Additional file 1. Figure S1 [file 12864_2023_9175_MOESM1_ESM.pdf]

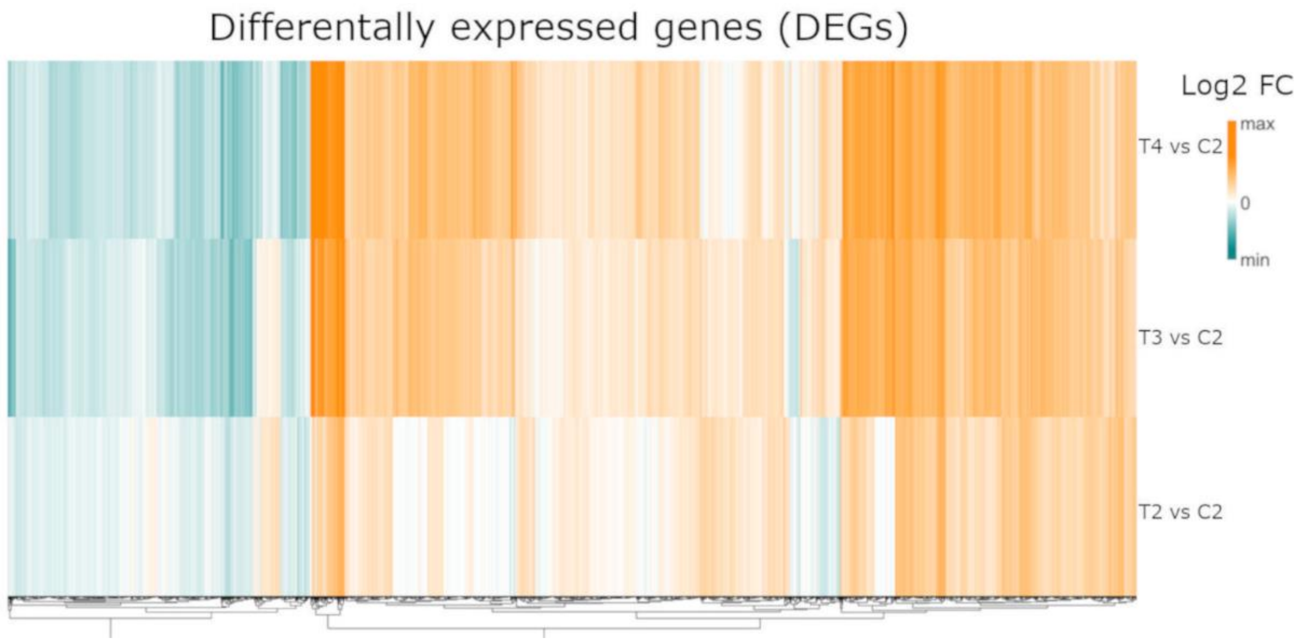

Figure S1: Hierarchical clustering analysis of log2(FC)s expression. The heatmap represents genes displaying statistical significance (log2(FC) ≥ 1.5, adjusted P-value ≤ 0.05) for differential expression between treatments (T2, T3, T4) versus control (C2). The up-regulated genes are reported in orange, the down-regulated genes in blue, and the genes not differentially expressed in white.
