# Supplementary material for: Gene expression during the formation of resting spores induced by nitrogen starvation in the marine diatom Chaetoceros socialis
Source: BMC Genomics. 2023 Mar 10;24:106. doi: 10.1186/s12864-023-09175-x (PMC9999646; doi:10.1186/s12864-023-09175-x)
Supplement: Supplementary file 2 — Additional file 2. Figure S2 [file 12864_2023_9175_MOESM2_ESM.pdf]

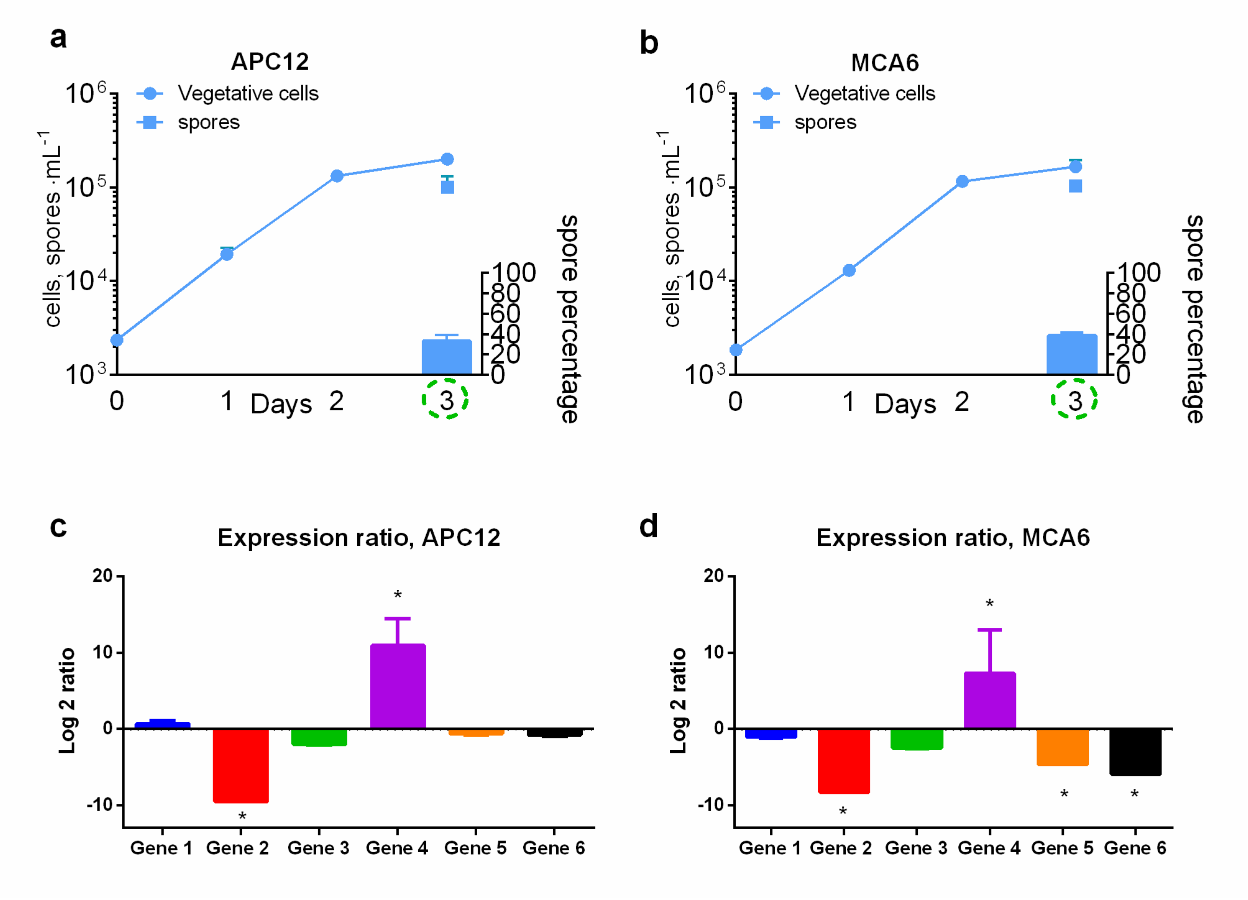

Figure S2: DEGs of *Chaetoceros socialis* validated with RT-qPCR. Cell and spore concentration (cells, spores mL^{-1}; left axis) and spore percentage (right axis) in strains APC12 (**a**) and MCA6 (**b**) grown under nitrogen starvation; dashed green circles mark the sampling point for the RT-qPCR analysis. The resulting gene expression (log2(FC)) for APC12 (**c**) and MCA6 (**d**). Asterisks mark statistically significant results ($P \leq 0.05$) of a pairwise fixed reallocation randomisation test. Gene 1: Cyclin-dependent kinase 5 homolog; gene 2: High affinity nitrate transporter 2.6; gene 3: Nitrate reductase; gene 4: Silicon efflux transporter; gene 5: Unknown; gene 6: 20 kDa chaperonin, chloroplastic (See Table S10 for their log2(FC) in the RNA-seq experiment).
