# Supplementary material for: Gene expression during the formation of resting spores induced by nitrogen starvation in the marine diatom Chaetoceros socialis
Source: BMC Genomics. 2023 Mar 10;24:106. doi: 10.1186/s12864-023-09175-x (PMC9999646; doi:10.1186/s12864-023-09175-x)
Supplement: Supplementary file 4 — Additional file 4. Figure S4 [file 12864_2023_9175_MOESM4_ESM.pdf]

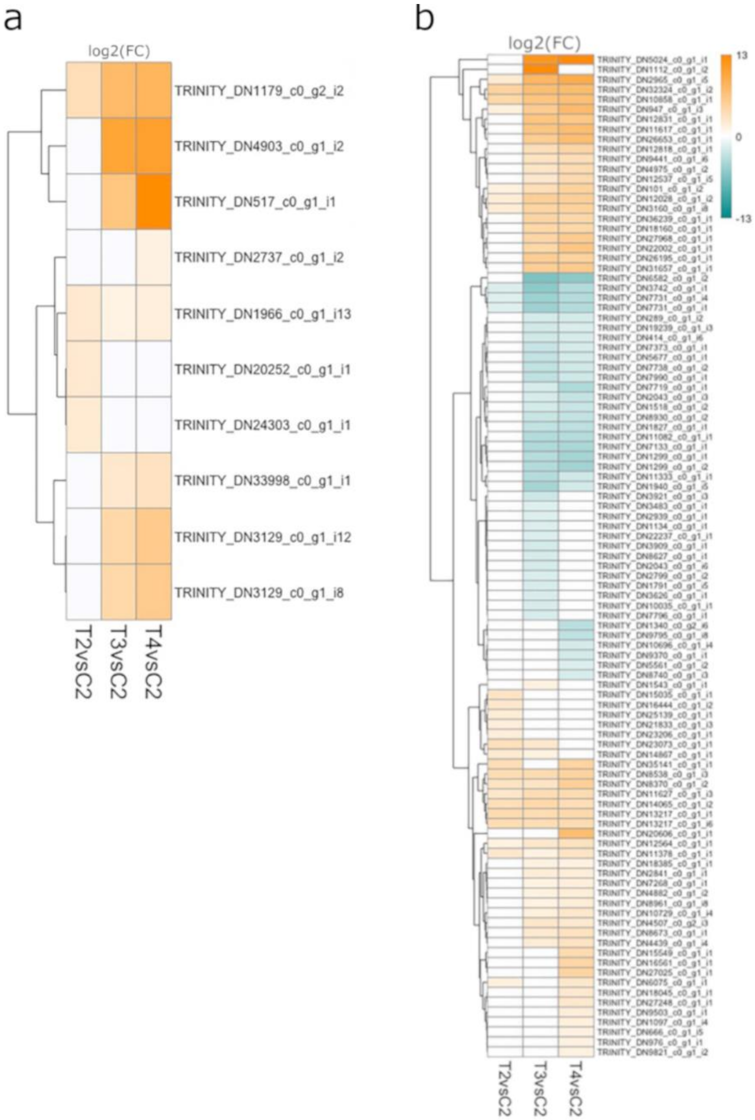

Figure S4: Hierarchical clustering analysis of FCs expression related to chemical signaling: oxylipin-mediated signalling (**a**) ROS production and programmed cell death (**b**). The heatmaps represent genes displaying statistical significance (log2(FC) $\geq$ 1.5, adjusted P-value $\leq$ 0.05) for differential expression between sampling points. The upregulated genes are reported in orange, the downregulated genes in blue and the not differentially expressed ones in white.
