# Supplementary material for: Gene expression during the formation of resting spores induced by nitrogen starvation in the marine diatom Chaetoceros socialis
Source: BMC Genomics. 2023 Mar 10;24:106. doi: 10.1186/s12864-023-09175-x (PMC9999646; doi:10.1186/s12864-023-09175-x)
Supplement: Supplementary file 6 — Additional file 6. Figure S6 [file 12864_2023_9175_MOESM6_ESM.pdf]

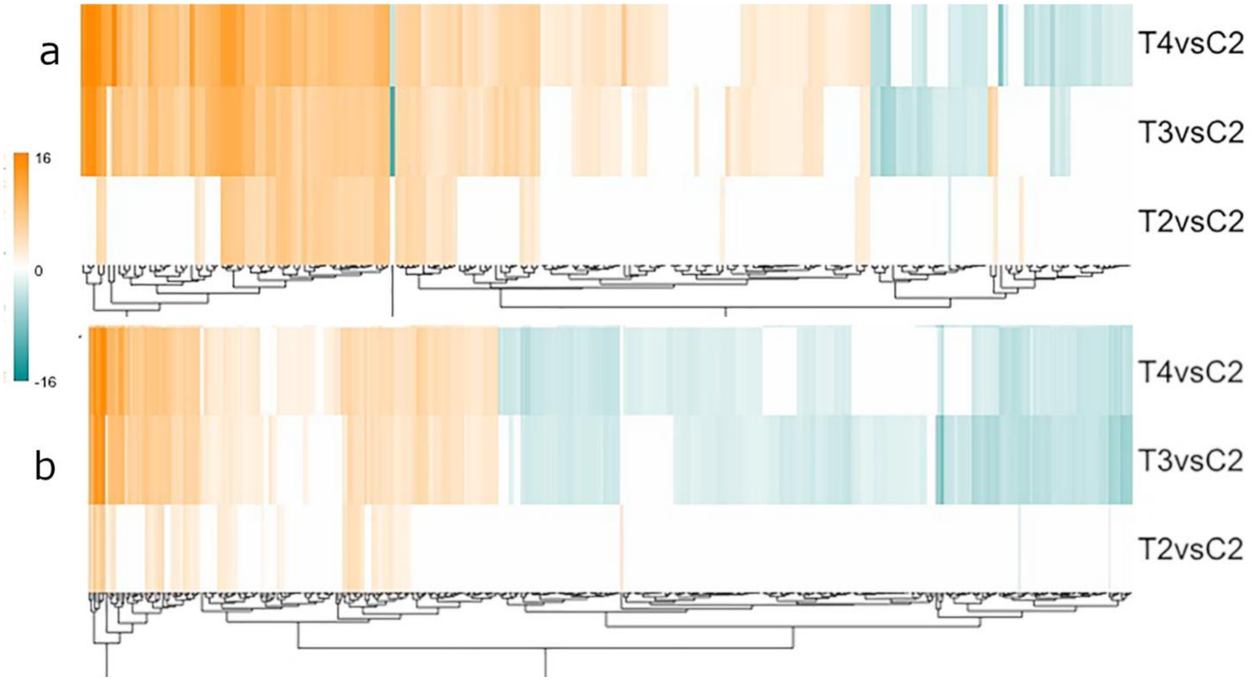

Figure S6: Hierarchical clustering analysis of FCs expression of *T. pseudonana* orthologs shared with *C. socialis*. The expression trend of upregulated (**a**) and downregulated (**b**) genes in *C. socialis* counterparts. The heatmap represents genes displaying statistical significance (log2(FC) ≥ 1.5, adjusted P-value ≤ 0.05) for differential expression between sampling points. The upregulated genes are reported in orange, the downregulated genes in blue and the not differentially expressed genes in white.
